# Angiosperm-like pollen and *Afropollis* from the Middle Triassic (Anisian) of the Germanic Basin (Northern Switzerland)

**DOI:** 10.3389/fpls.2013.00344

**Published:** 2013-10-01

**Authors:** Peter A. Hochuli, Susanne Feist-Burkhardt

**Affiliations:** ^1^Palaeontological Institute and Museum, University of ZürichZürich, Switzerland; ^2^Dr. Susanne Feist-Burkhardt Geological Consulting & ServicesOber-Ramstadt, Germany

**Keywords:** Middle Triassic, angiosperm-like pollen, angiosperm stem group, *Afropollis*, confocal laser scanning microscopy

## Abstract

Here we report on angiosperm-like pollen and *Afropollis* from the Anisian (Middle Triassic, 247.2–242.0 Ma) of a mid-latitudinal site in Northern Switzerland. Small monosulcate pollen grains with typical reticulate (semitectate) sculpture, columellate structure of the sexine and thin nexine show close similarities to early angiosperm pollen known from the Early Cretaceous. However, they differ in their extremely thin inner layer (nexine). Six different pollen types (I–VI) are differentiated based on size, reticulation pattern, and exine structure. The described pollen grains show all the essential features of angiosperm pollen. However, considering the lack of a continuous record throughout the lower part of the Mesozoic and the comparison with the oldest Cretaceous finds we suggest an affinity to an angiosperm stem group. Together with the previously published records from the Middle Triassic of the Barents Sea area the angiosperm-like pollen grains reflect a considerable diversity of the parent plants during the Middle Triassic. Sedimentological evidence and associated palynofloras also suggest a remarkable ecological range for these plants. Associated with these grains we found pollen comparable to the genus *Afropollis*. Representatives of this genus are commonly recorded in Lower Cretaceous sediments of low latitudes, but until now had no record from the lower part of the Mesozoic.

## Introduction

In spite of extensive research the origin and temporal and spatial distribution of early flowering plants are still a matter of debate. Current research on the origin of angiosperms follows two lines of evidence—the fossil record and molecular data. Molecular data provide a framework for the origin of the crown group of flowering plants as well as for the successive appearance and relationships of the various clades. A wealth of molecular data has been published over the last two decades (for references see Frohlich and Chase, 2007; Soltis et al., 2010, 2011; Doyle, 2012). However, so far no consensus about the first appearance of the angiosperms has been reached. Different data as well as methods have resulted in significantly different ages ranging from the Palaeozoic to the Cretaceous (e.g., Bell et al., 2010; Magallón, 2010; Smith et al., 2010; for a recent overview see Doyle, 2012 and Magallón et al., 2013).

In this paper we focus on fossil evidence, presenting the so far oldest angiosperm-like pollen from the Middle Triassic (ca. 243 Ma), a record that predates the generally accepted first occurrence of angiosperm pollen by more than 100 Ma. The first known fossil angiosperm pollen grains as well as those of the most basal living species are described as columellate and monosulcate. That means they have one distal slit-like aperture (sulcus) and a wall structure including column like elements (columellae). The multilayer wall (exine), which is commonly preserved in the fossil record, consists of the thin innermost endexine followed by an adjacent thin layer, termed footlayer. The following columellate layer, consisting of rod like elements, roots in the footlayer and connects it to the outermost layer called tectum. Since endexine and footlayer are commonly indistinguishable—especially in light microscope studies—the term nexine has been introduced. The term sexine is used to designate the outer layer consisting of the columellate layer and the tectum. In the present study the term nexine is consistently applied for the inner layer, since with the presently used optical means endexine and footlayer cannot be differentiated. The tectum of the above mentioned pollen grains is commonly discontinuous or perforated, which is described with the term semitectate. Thus, the complex morphology of the exine of angiosperm pollen permits to distinguish them from superficially similar palynomorphs, like gymnosperm pollen, some spores, dinoflagellate cysts or other algal and fungal remains.

In the present study we discuss finds of angiosperm-like, monosulcate and columellate pollen grains in the context of the earliest Cretaceous and other pre-Cretaceous records. Early Cretaceous diversification and evolutionary success of flowering plants is well documented by numerous finds of pollen and megafossils—such as flowers, wood, seeds, and leaves. The earliest accepted records are essentially based on dispersed pollen grains, which due to their high numbers and their high preservation potential represent the most appropriate tool to trace the timing of early angiosperm evolution. The presence of angiosperm pollen in biostratigraphically dated marine sediments provides the possibility to precisely calibrate the essential steps in angiosperm evolution (Muller, 1981; Hughes, 1994; Heimhofer et al., 2005; Doyle, 2012).

The first broadly accepted records of angiospermous pollen grains are known from the earlier part of the Early Cretaceous (Valanginian—early Hauterivian) (Gübeli et al., 1984; Trevisan, 1988; Brenner, 1996). However, within this interval they are extremely rare; continuous records exist only from the Barremian onwards, and this only for low and mid-latitudes (Hickey and Doyle, 1977; Crane and Lidgard, 1989; Hughes, 1994; Schrank and Mahmoud, 2002). Based on their subsequent increase in abundance and diversity it has been assumed that angiosperms originated in the Early Cretaceous (e.g., Hughes, 1994; Brenner, 1996; Friis et al., 2011). However, some authors suggest that the “sudden appearance” on most continents as well as the rapid radiation of numerous clades during the latter part of the Early Cretaceous point to an earlier origin of the group, probably going back to the Jurassic (e.g., Stuessy, 2004; Doyle, 2012), to the Triassic or even to the Palaeozoic (Zavada, 2007; Clarke et al., 2011). Cornet (1977, 1989b) was the first who reported on occurrences of pollen grains with angiospermous features from Triassic sediments. However, these records are commonly regarded as inadequate evidence for an earlier origin of the group (e.g., Friis et al., 2011). But so far the search for unequivocal evidence (fossil flowers and carpels) from pre-Cretaceous sediments was unsuccessful. Either the age of the sediment containing larger plant remains was erroneous (Sun et al., 1998; He et al., 2004, 2006) or the plant fossils were not sufficiently well preserved to allow for unambiguous interpretation (Wang et al., 2007; Wang, 2009, 2010; Zheng and Wang, 2010; Friis et al., 2011; Doyle, 2012). From the same area as the angiosperm-like pollen grains we report on the oldest occurrence of the genus *Afropollis*. This group, considered by some authors as of angiospermous affinity, is so far known to be restricted to the Cretaceous (Barremian–Cenomanian). Its presence in Middle Triassic sediments opens another observation gap of over 100 Ma.

## Location and stratigraphic context

**Figure 1:**
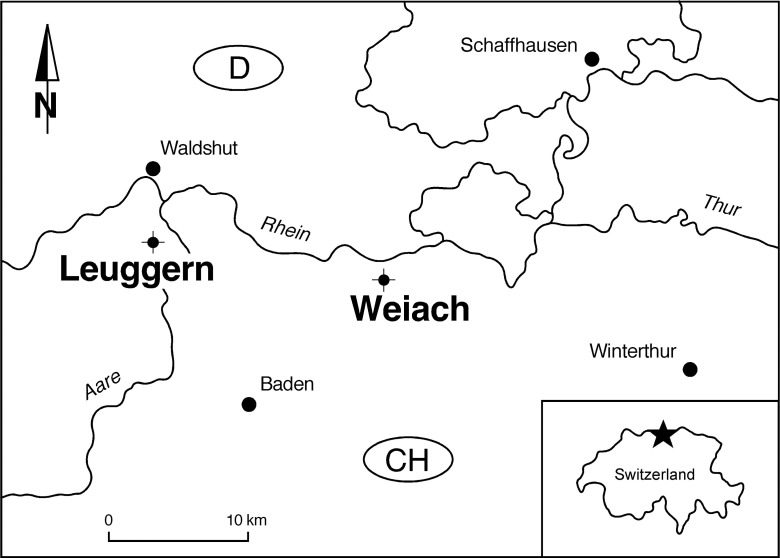
**Location of the Weiach and Leuggern core holes**.

**Figure 2:**
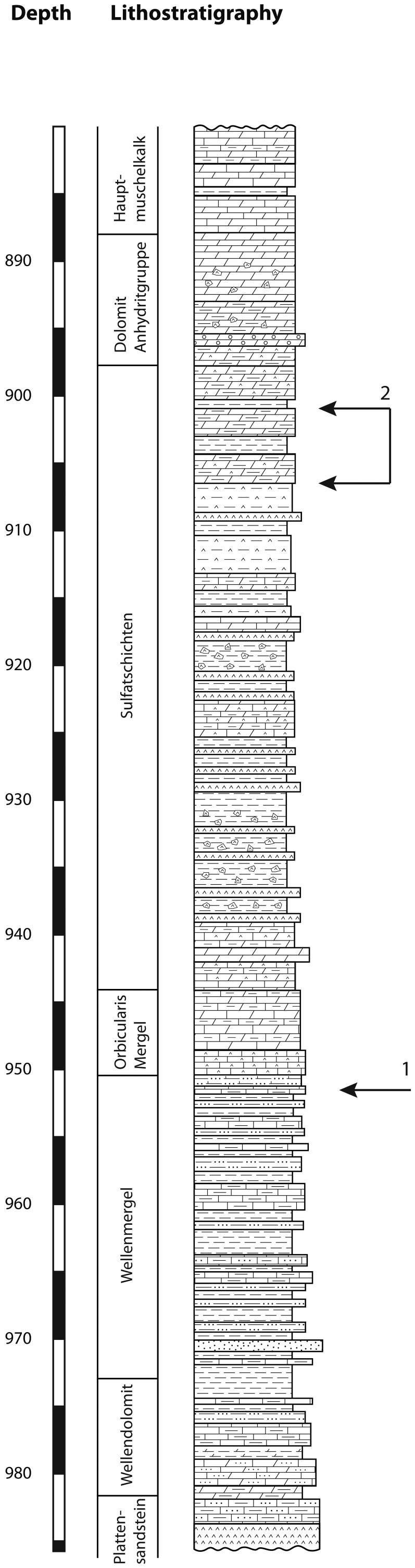
**Lithostratigraphic column of the Middle Triassic section of the Weiach core hole.** 1, position of *Afropollis*; 2, range of angiosperm-like pollen grains.

The illustrated pollen grains have been observed in samples from two core holes in Northern Switzerland (Weiach and Leuggern, Figure 1). During the Middle Triassic the site in Northern Switzerland was located at a palaeolatitude of about 20°N. A continuous Middle Triassic section was recovered from the Weiach core hole (Matter et al., 1988, and Figure 2). The studied interval includes the uppermost part of the Buntsandstein (Plattensandstein), the lower part of the Muschelkalk (Wellenkalk/Wellenmergel) and the middle part of the Muschelkalk (Sulfatschichten) representing a typical succession for the southern part of the Germanic Basin (for geological details see Figure 2 as well as Matter et al., 1988; Peters et al., 1989).

The described pollen grains have been observed at two distinct levels of the Weiach core hole (Figure 2). The sample containing *Afropollis* comes from the Wellenkalk unit (Lower Muschelkalk), which consists essentially of gray siltstones. The associated palynological assemblage consists of typical elements of the early Anisian (Aegean) including *Stellapollenites thiergartii, Tsugaepollenites oriens*, *Densoisporites nejburgii*, and *Platysaccus leschikii* (see Kürschner and Herngreen, 2010; Hochuli et al., 2012). Another record of *Afropollis* (*Afropollis* sp. II) has been found in the same unit of the neighboring Leuggern core hole.

The occurrence of the angiosperm-like pollen is restricted to the upper part of the Sulfatschichten (Middle Muschelkalk), which is dominated by evaporites. The associated palynoflora consists also of typical Anisian taxa including *Stellapollenites thiergartii, Tsugaepollenites oriens*, *Podosporites amicus*, *Institisporites* spp., as well as frequent occurrence of *Protodiploxypinus* and a few representatives of the Circumpolles group (e.g., *Duplicisporites tenebrosus* and *Paracirculina* spp.) (Hochuli et al., 2012). Dominating elements are conifer pollen of the *Triadispora* group whereas spores are rare. This assemblage is interpreted to be of middle to late Anisian (Pelsonian/Illyrian) age (see Kürschner and Herngreen, 2010; Hochuli et al., 2012).

## Materials and methods

*Afropollis* has been found in the fine-grained dominantly siliciclastic Wellenkalk unit (Lower Muschelkalk) of the Weiach core hole (950.82 m) and in the same unit of the nearby Leuggern core hole (171.63 m). The angiosperm-like pollen grains have been found in a short interval (901.91–905.99 m) of the Sulfatschichten of the Weiach core hole. The samples originate from interbedded gray silty layers in this evaporite dominated interval. The described angiosperm-like pollen grains are very rare, representing <1% of the pollen counts. In the studied sections palynomorphs are well preserved and show no sign of thermal alteration (Thermal Alteration Index <2; Staplin, 1982); however, they are strongly compressed. Contamination of the samples can be excluded since recent pollen grains would be three-dimensionally preserved and the described pollen types differ from superficially similar grains from Cretaceous and younger sediments.

The samples were processed according to standard palynological procedures (Traverse, 2007). The slides were produced by embedding the organic residues in epoxy resin. They were studied first using a standard microscope with transmitted light (Olympus BX51). Light microscope images were taken with an Olympus digital camera (Olympus DP12). Subsequently the described pollen grains were analyzed using confocal laser scanning microscopy (CLSM). CLSM is an optical imaging technique allowing for the capture of high resolution, blur-free fluorescence images and image stacks of optical sections through an embedded specimen. It enables the reconstruction of three-dimensional structures from the obtained image stacks. The technique is non-destructive and particularly suited to the analysis of palynomorphs. Because of the autofluorescence of most organic-walled microfossils standard palynological slides can be analyzed without any further preparation. Its application to fossil organic-walled dinoflagellate cysts was first described in detail by Feist-Burkhardt and Pross (1999). Hochuli and Feist-Burkhardt (2004) used CLSM in the morphological analysis of Triassic angiosperm-like pollen grains. In this paper we show some associated gymnospermous pollen grains for comparison. The confocal laser scanning images show the distinct differences in the structure of the pollen grains. Confocal images were taken on a Leica TCS SP confocal microscope using 40x or 100x objectives under oil immersion at an excitation wavelength of 488 nm and detection of emitted fluorescence light at 500 nm and longer. Optical sections were captured with an image resolution of 1024 × 1024 pixels and a distance between sections of less than 400 nm. The resulting image stacks were then further processed using the Open Source image processing package FIJI on a Mac OSX computer (http://fiji.sc/Fiji, Schindelin et al., 2012).

## Results/descriptions

Below we describe six distinct monosulcate, semitectate pollen grains (type I–VI). They are all characterized by reticulate sculpture and columellate connections to an extremely thin nexine. The applied CLSM technique provides detailed images of the surface of the pollen grains and evidence for the structuring of the exine (see Plate I–IV). However, with the applied method it is impossible to differentiate endexine and footlayer. The six pollen types are differentiated based on their size, the development of the reticulum and the shape of the sulcus. So far only a few grains or single specimens are available for each type; for this reason we refrain from describing formal species. Additionally, we describe two pollen grains that we attribute to the genus *Afropollis* (*Afropollis* sp. I and II, see Plate IV). They show the characteristic reticulum of this genus, but—as in many Cretaceous forms—the inner wall (nexine) is missing or extremely shrunken.

**Plate I:**
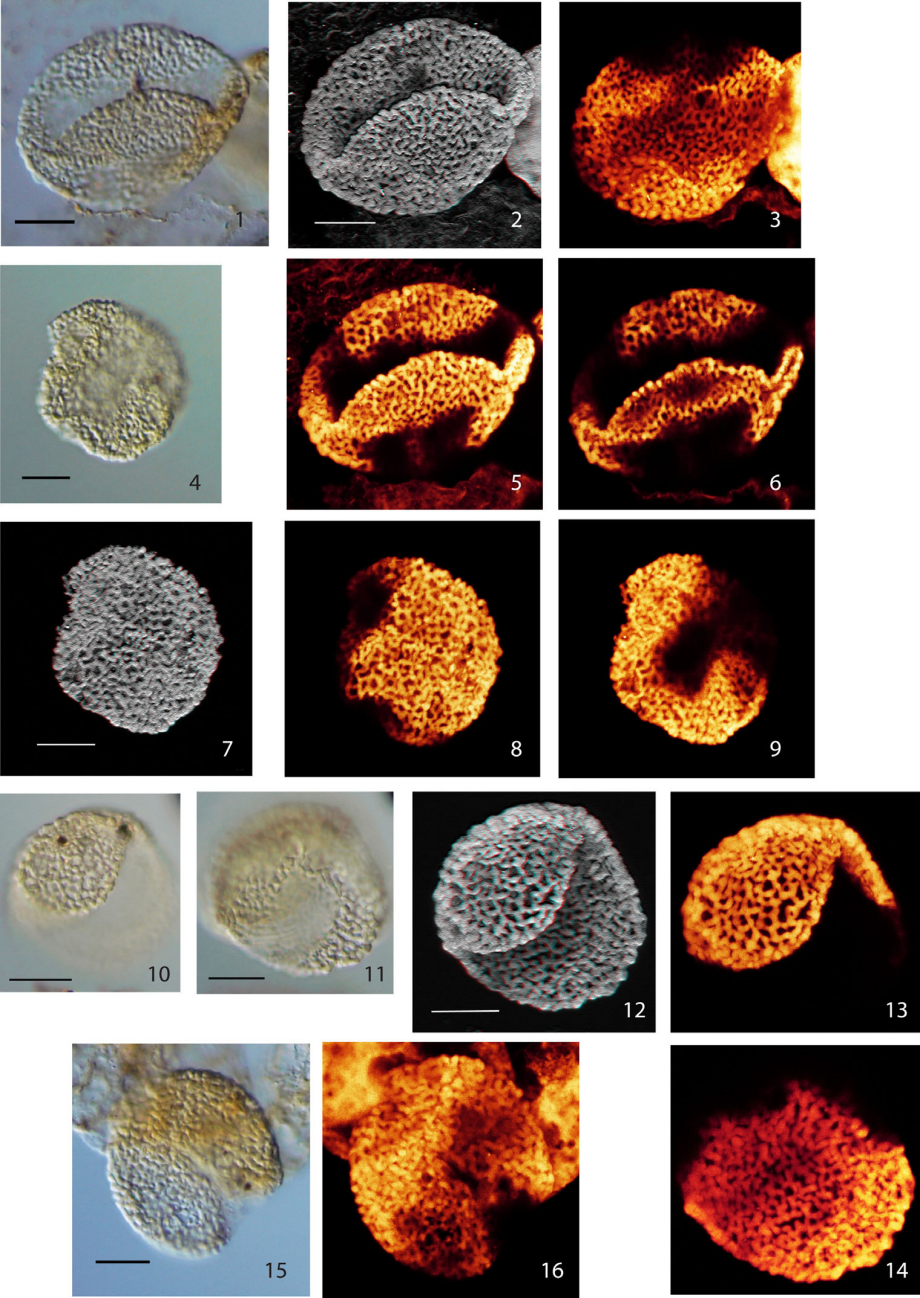
**Scale bars: 10 μm. (1)**, Pollen Type I, specimen A, LM image (high focus); **(2)**, Pollen Type I, specimen A, CLSM image, total stack image/projection, anaglyph; **(3)**, Pollen Type I, specimen A, CLSM partial stack image, proximal side; **(4)**, Pollen Type I, specimen B, LM image (median focus); **(5)**, Pollen Type I, specimen A, CLSM partial stack image, distal side; **(6)**, Pollen Type I, specimen A, CLSM single image, optical section; **(7)**, Pollen Type I, specimen B, CLSM total stack image/projection, anaglyph; **(8)**, Pollen Type I, specimen B, CLSM partial stack image, lateral view (high focus); **(9)**, Pollen Type I, specimen B, CLSM partial stack image, lateral view (low focus); **(10)**, Pollen Type II, specimen A, LM image, high focus; **(11)**, Pollen Type II, specimen A, LM image, low focus; **(12)**, Pollen Type II, specimen A, CLSM total stack image/projection, anaglyph; **(13)**, Pollen Type II, specimen A, CLSM partial stack image, lateral view on distal side (high focus); **(14)**, Pollen Type II, specimen A, CLSM partial stack image, lateral view on proximal side (low focus); **(15)**, Pollen Type II, specimen B, LM image, high focus; **(16)**, Pollen Type II, specimen B, CLSM total stack image/projection.

**Plate II:**
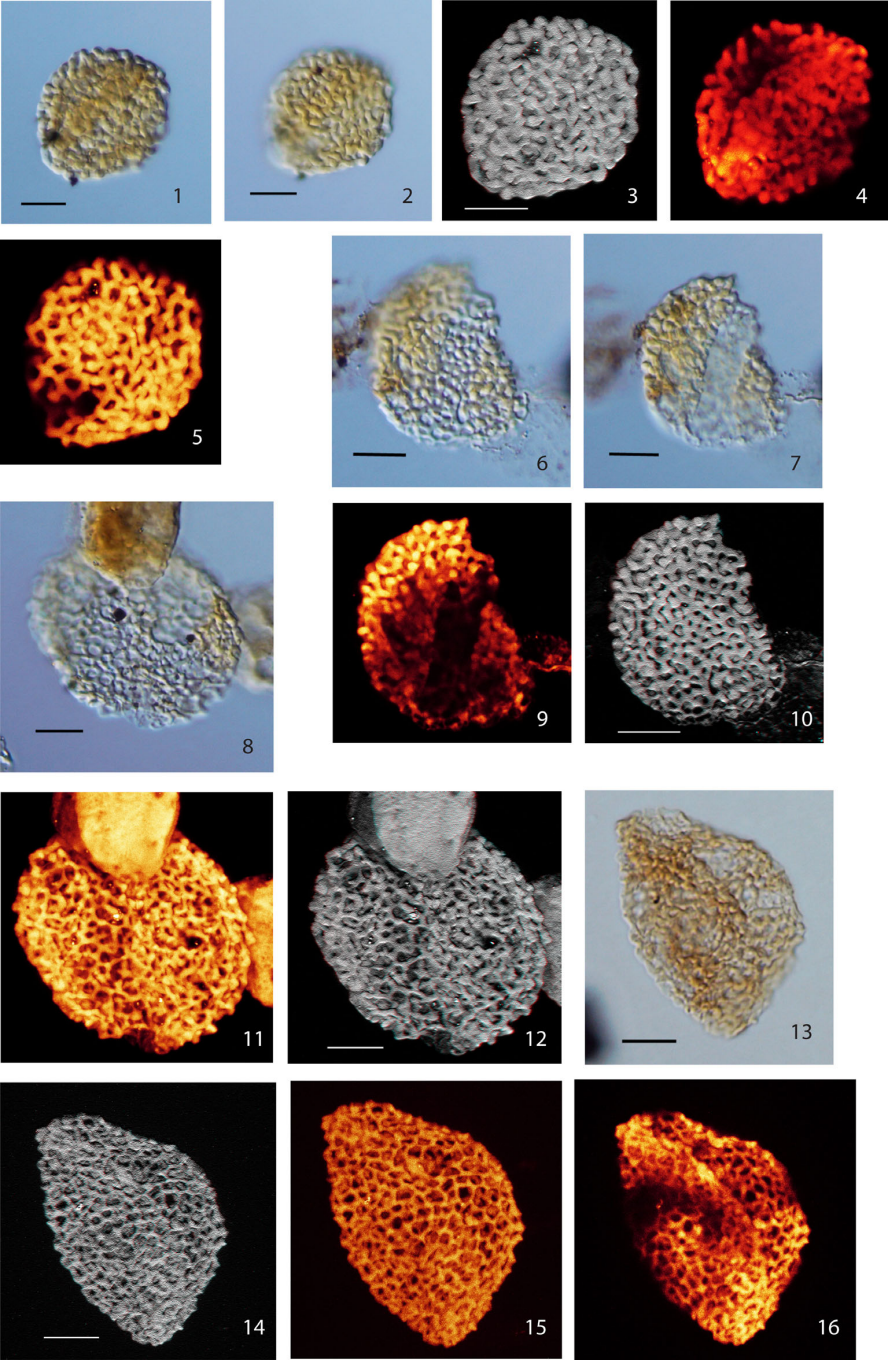
**Scale bars: 10 μm. (1)**, Pollen Type III, specimen A, LM image, median focus; **(2)**, Pollen Type III, specimen A, LM image, (high focus); **(3)**, Pollen Type III, specimen A, CLSM total stack image/projection, anaglyph; **(4)**, Pollen Type III, specimen A, CLSM partial stack image, distal side (low focus); **(5)**, Pollen Type III, specimen A, CLSM partial stack image, proximal side (high focus); **(6)**, Pollen Type III, specimen B, LM image (high focus); **(7)**, Pollen Type III, specimen B, LM image (low focus); **(8)**, Pollen Type IV, specimen A, LM image (high focus); **(9)**, Pollen Type III, specimen B, CLSM partial stack image, distal side (low focus); **(10)** Pollen Type III, specimen B, CLSM total stack image/projection, anaglyph; **(11)**, Pollen Type IV, specimen A, CLSM total stack image/projection; **(12)**, Pollen Type IV, specimen A, CLSM total stack image/projection, anaglyph; **(13)**, Pollen Type IV, specimen B, LM image (median focus); **(14)** Pollen Type IV, specimen B, CLSM total stack image/projection, anaglyph; **(15)** Pollen Type IV, specimen B, CLSM total stack image/projection; **(16)** Pollen Type IV, specimen B, CLSM partial stack image, distal side (median focus).

**Plate III:**
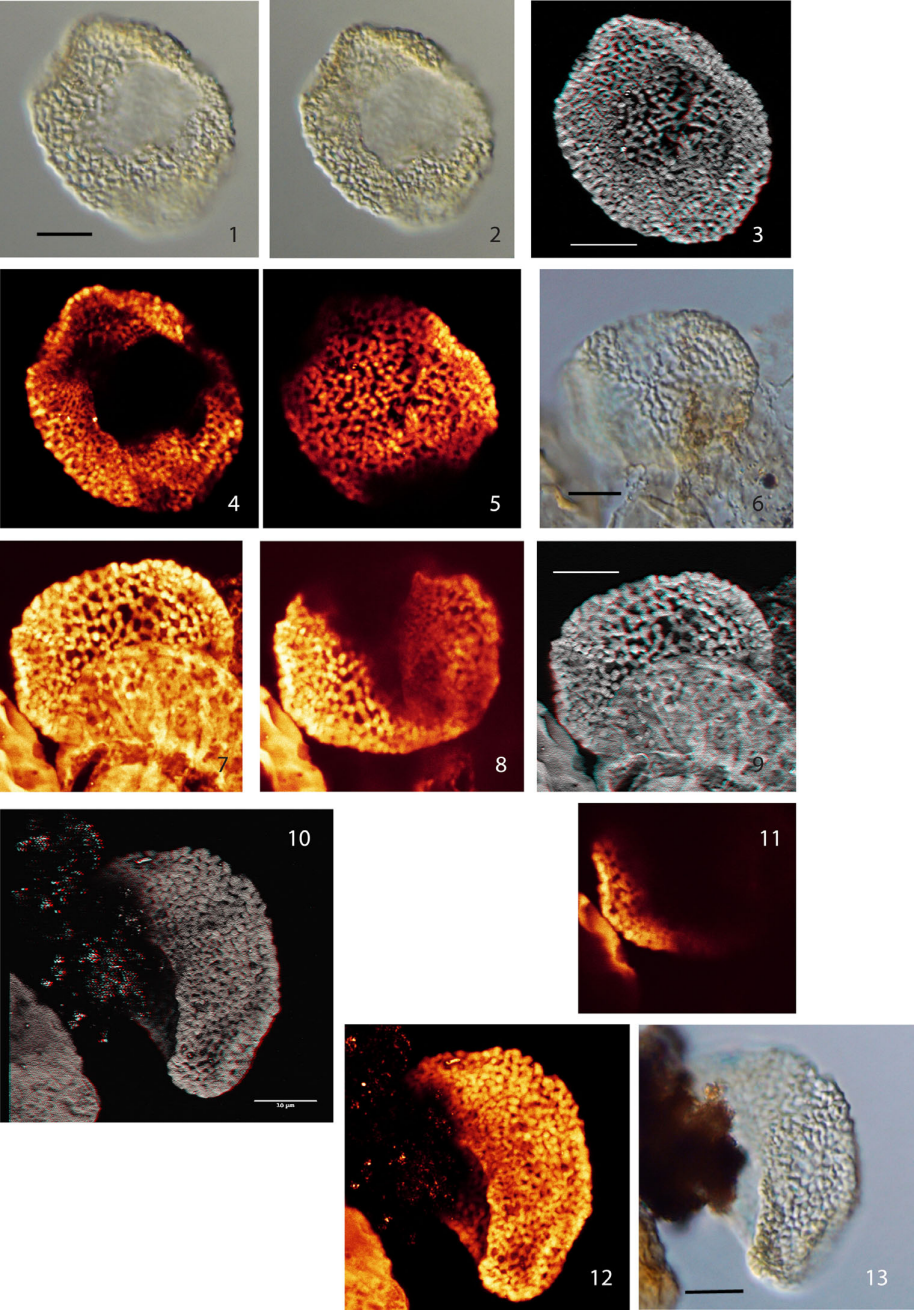
**Scale bars: 10 μm. (1)**, Pollen Type V, specimen A, LM image (median focus); **(2)**, Pollen Type V, specimen A, LM image (high focus); **(3)** Pollen Type V, specimen A, CLSM total stack image/projection, anaglyph; **(4)** Pollen Type V, specimen A, CLSM partial stack image, distal side (high focus); **(5)** Pollen Type V, specimen A, CLSM partial stack image, proximal side (low focus); **(6)** Pollen Type V, specimen B, LM image (high focus); **(7)** Pollen Type V, specimen B, CLSM total stack image/projection; **(8)** Pollen Type V, specimen B, CLSM partial stack image, lateral view on distal side (low focus); **(9)** Pollen Type V, specimen B, CLSM total stack image/projection, anaglyph; **(10)** Pollen Type V, specimen C, CLSM total stack image/projection, anaglyph, lateral view; **(11)**, Pollen Type V, specimen B, CLSM single image, optical section; **(12)**, Pollen Type V, specimen C, CLSM total stack image/projection, lateral view; **(13)**, Pollen Type V, specimen C, LM image, lateral view (high focus).

**Plate IV:**
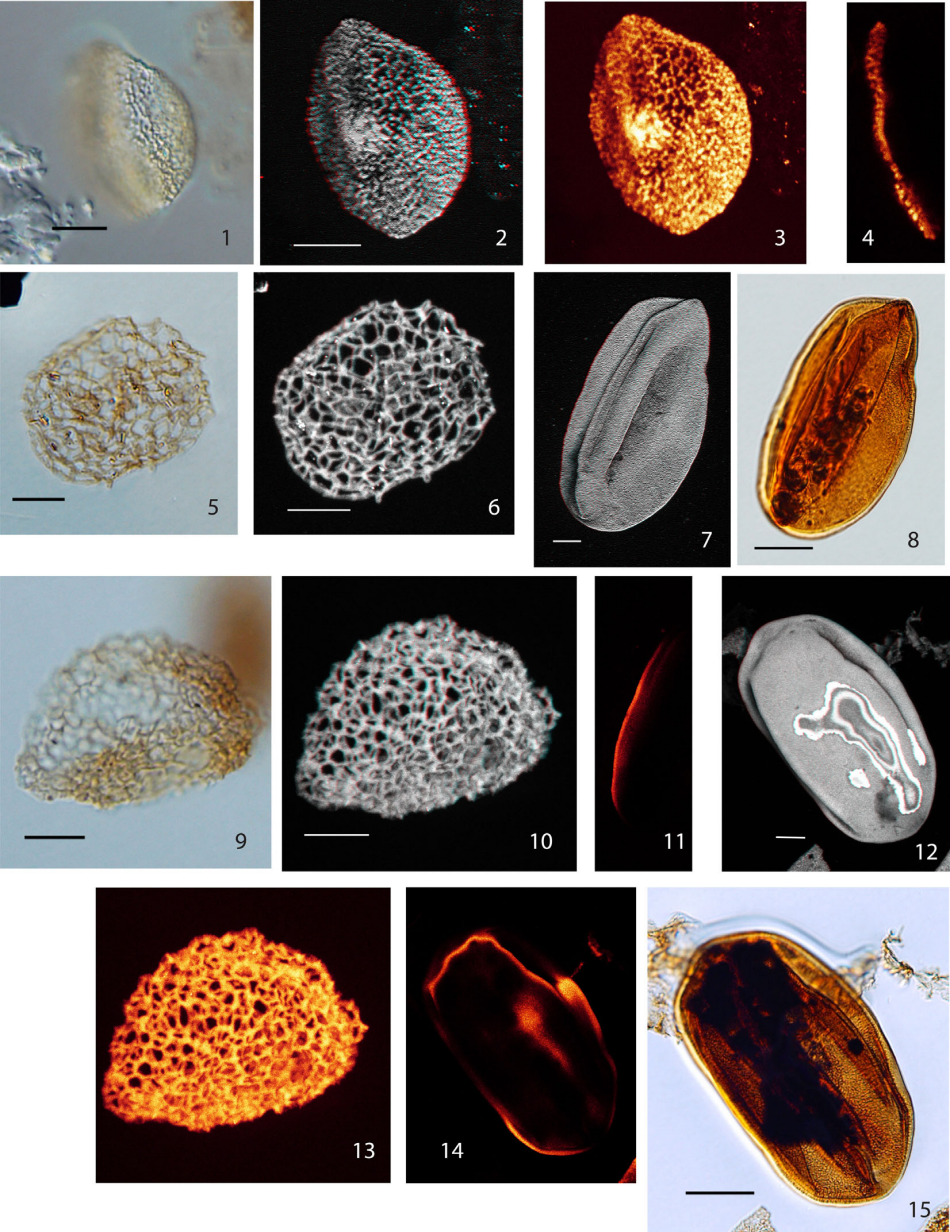
**Scale bars: 10 μm**, except **(8)** and **(15)** (20 μm); **(1)** Pollen Type VI, LM image (high focus); **(2)** Pollen Type VI, CLSM total stack image/projection, anaglyph; **(3)**, Pollen Type VI, CLSM total stack image/projection; **(4)**, Pollen Type VI, CLSM single image, optical section; **(5)**, *Afropollis* sp. I, LM image (median focus); **(6)** *Afropollis* sp. I, CLSM total stack image/projection, anaglyph; **(7)** *Eucommiidites* sp. 1, CLSM total stack image/projection, anaglyph; **(8)**, *Eucommiidites* sp. 1, LM image (median focus); **(9)** *Afropollis* sp. II, LM image, high focus; **(10)**, *Afropollis* sp. II, CLSM total stack image/projection, anaglyph; **(11)**, *Eucommiidites* sp. 1, CLSM single image, optical section; **(12)**, *Eucommiidites* sp. 2, CLSM total stack image/projection, anaglyph; **(13)**, *Afropollis* sp. II, CLSM total stack image/projection; **(14)**, *Eucommiidites* sp. 2, CLSM single image, optical section; **(15)**, *Eucommiidites* sp. 2, LM image (median focus).

For comparison and to demonstrate the distinct differences in the optical characteristics we document two pollen grains comparable to the gymnosperm pollen genus *Eucommiidites* from the same sections (Plate IV, 7, 8, 11, 12, 14, 15). The resolution of the CLSM images shows their homogeneous or slightly granular wall structure, representing a distinct contrast to the exine structure of the columellate, angiosperm-like grains.

Positions on the slides are indicated in England-Finder coordinates.

### Type I

**Material: 2 specimens; specimen A: Weiach 903.02 m, N38; specimen B: Weiach 901.91 m, T59**

Specimen A: Plate I, (1–3, 5, 6); specimen B: Plate I, (4, 7–9)

**Description:** Monosulcate, columellate, semitectate, densely reticulate pollen grains

Size: specimen A: 31.5 × 41.5 μm; specimen B: 33.0 × 32.0 μm

Shape: oval to nearly spherical, compressed

Sulcus: broad open (folded in specimen A)

Wall/structure: ca. 0.7–1.0 μm thick; columellae: 0.5–1.5 μm (globular); nexine very thin.

Sculpture: reticulate, slightly heterobrochate, smallest meshes near the sulcus and towards the end of the sulcal area. Shape of meshes irregular; mesh size: 0.5–2.0 μm. Muri: winding, beaded, width 0.5–1.5 μm (−2.0 μm, columellae head), thickened at the vertices of the reticulum, surface with flat verrucae resulting from the slightly protruding heads of columellae.

### Type II

**Material:** 2 specimens; specimen A: Weiach 903.02 m, G63/3; specimen B: Weiach 901.91 m, T58/3

Specimen A: Plate I, (10–14); specimen B: Plate I, (15, 16)

**Description:** Monosulcate, columellate, semitectate, reticulate pollen grains

Size: specimen A: 30.0 × 32.0 μm/specimen B: 30.0 × 35.0 μm

Shape: spherical, compressed

Sulcus: short open slit (see Type I)

Wall/structure: 0.5–1.0 μm thick (−1.5 μm, head of columellae), columellate. Columellae club-shaped to almost spherical (0.6–1.5 μm) arranged in a reticulate pattern, reduced in size toward the sulcus (Plate I, 11, 12); nexine very thin.

Sculpture: irregularly reticulate, heterobrochate, meshes with irregular shape and size; size: 0.5–3.5 μm. Muri: consisting of columellae arranged in a reticulate pattern; width 0.5–2.5 μm (head of columellae); slightly beaded, surface with flat verrucae (protruding heads of columellae).

### Type III

**Material**: 2 specimens; specimen A: Weiach 901.91 m, Q37/4; specimen B: Weiach 901.91 m, U64/2

Specimen A: Plate II, (1–5); specimen B: Plate II, (6, 7, 9, 10)

**Description:** Monosulcate, columellate, semitectate, reticulate pollen grains

Size: 28.0 × 32.0 μm/26 × 35.0 μm

Shape: spherical to ellipsoidal, compressed

Sulcus: slit-like or broad, open

Wall/structure: 0.5–2.0 μm thick, columellate; columellae club-shaped to nearly globular, most pronounced at the vertices of the muri; nexine very thin.

Sculpture: reticulate, heterobrochate; reticulum finer towards sulcus; meshes mostly roundish, mesh size 0.8–3.2 μm. Muri: coarse, width: (0.5 –) 1.0–1.5 μm; surface verrucate as a result of protruding heads of columellae.

### Type IV

**Material:** 2 specimens; specimen A: Weiach 901.91 m, J40/3; specimen B: Weiach 950.82 m, V55/1

Specimen A: Plate II, (8, 11, 12); specimen B, Plate II, (13–16)

**Description:** Monosulcate, columellate, semitectate, reticulate, heterobrochate pollen grains

Size: specimen A: 40 × 47.0 μm/specimen B: 32.0 × 46.0 μm

Shape: spherical–ellipsoidal, compressed

Sulcus: slit-like, elongate, narrow

Wall/structure: 2.0 μm thick, columellate, columellae: ca. 2.0 μm, nearly spherical, head diameter: 1.0–2.2 μm; nexine very thin.

Sculpture: reticulate, heterobrochate, reduced lumina near the sulcus; lumina mostly roundish; mesh size: 1.0–3.0 μm. Muri: width 0.5–1.5 μm (max. 2.2 μm, head of columellae), vertices slightly thickened; surface: verrucate resulting from protruding heads of columellae at the vertices of the reticulum.

### Type V

**Material:** 3 specimens; specimen A: Weiach 901.91 m, G53; specimen B: Weiach 903.02 m, M52/1; specimen C: Weiach 901.91 m, H40/1

Plate III, (1–5) (specimen A); Plate III, (6–9, 11) (specimen B); Plate III, (10, 12, 13) (specimen C)

**Description:** Monosulcate, columellate, semitectate, finely reticulate to perforate pollen grains

Size: specimen A: 34.0 × 41.0 μm, specimen B: 29 × 36 μm, specimen C: 27.0 × 42.0 μm

Shape: nearly spherical–ellipsoidal (boat shaped), compressed

Sulcus: round–broad elongate, open

Wall/structure: 0.5–1.0 μm (−1.3 μm including columellae); columellate, columellae nearly spherical–club-shaped: 0.5–1.3 μm high, head diameter: 0.5–1.5, 0.2 μm near sulcus; nexine very thin.

Sculpture: reticulate, heterobrochate, mesh size reduced to perforate toward sulcus; mesh size: 1.2–2.4 μm (proximal); 0.2–1.9 μm (distal). Muri forming a reticulate pattern; width: 0.5–1.0 μm (0.3 um distally and 1.5 μm at max. head diameter); surface verrucate formed by prominent columellae heads.

### Type VI

**Material:** 1 specimen: Weiach 905.99 m, Y49/1

Plate IV, (1–4)

**Description:** Monosulcate, columellate, finely irregularly reticulate pollen grain

Size: 22.0 × 34.0 μm

Shape: ellipsoidal, boat shaped, compressed

Sulcus: narrow slit?

Wall/structure: 0.5 μm thick, semi-intectate, columellate. Tectum is reduced to few connections between the columellae. Columellae roundish to club-shaped, 0.5–0.8 μm high; nexine very thin.

Sculpture: fine, irregularly reticulate pattern. Mesh size 0.5–1.8 μm. Muri: 0.3–0.6 μm wide (columellae heads −0.8 μm); surface finely verrucate resulting from the protruding ends of columellae.

#### Afropollis sp. I

**Material:** 1 specimen: Weiach 950.82 m, U53/4

Plate IV, (5, 6)

**Description:** Monosulcate/inaperturate, coarsely reticulate pollen grain.

Size: 44.0 × 30.0 μm

Shape: ellipsoidal, compressed, probably in lateral view

Sulcus: not observed

Wall/structure: only sexine recognizable, nexine—if present—thin and detached;

Sculpture: Reticulum heterobrochate, lumina roundish to polygonal, mesh size: 1.0–3.5 μm; columellae not visible. Muri higher than wide, width ca. 0.5 μm, ca. 1.0 μm high, with radial elements; surface echinate to verrucate as a result of radially protruding elements. *Afropollis* sp. I differs from *Afropollis* sp. II and from most of the species described from the Cretaceous in having more regular (not sinuous) muri.

#### Afropollis sp. II

**Material:** 1 specimen: Leuggern 171.63 m, R55

Plate IV, (9, 10, 13)

**Description:** Inaperturate, reticulate pollen grain

Shape: spherical, compressed, slightly folded and partly broken.

Size: 37.0 × 32.0 μm

Sulcus: not observed

Wall/structure: only sexine recognizable, nexine—if present—thin and detached.

Reticulum: homobrochate, lumina polygonal– roundish, and slightly protruding elements at the vertices of the reticulum, mesh size: 2.0–4.5 μm. Muri: partly sinuous, ca. 0.5 μm wide, 0.5–1.5 μm high, with dispersed radial elements, some of them few protruding.

This pollen grain resembles those reported as type G by Hochuli and Feist-Burkhardt (2004). However, on pollen Type G the radial elements are poorly expressed; for this reason it was not assigned to *Afropollis*.

### Gymnosperm pollen

#### Eucommiidites sp. 1

**Material:** Weiach 980.57 m, O59/1

Plate IV, (7–8, 11)

*Eucommiidites* sp. 1 has a granulate surface. The wall is densely granulate.

Size: 92 × 47 μm

#### Eucommiidites sp. 2

**Material:** Weiach 978.47 m; J52/2

Plate IV, (12, 14–15)

Size: 90 × 50 μm

## Discussion—cretaceous and pre-cretaceous records

Pollen grains are easily dispersed over wide areas due to their relatively small size and the high numbers produced. They can be found in various depositional environments (marine, coastal, and terrestrial). For this reason records of fossil pollen are most complete if compared to other plant organs such as seeds or leaves. Generally accepted first records of angiosperm pollen are mentioned from the early part of the Early Cretaceous (Valanginian—early Hauterivian), corresponding to an age range of approximately 139–133 Ma. However, within this interval they are extremely rare and poorly documented. Most palynological records from the basal Cretaceous (Berriasian–Valanginian) lack angiosperm pollen (e.g., Norris, 1969; Dörhöfer, 1977; Dörhöfer and Norris, 1977; Doyle, 1983; Regali and Viana, 1989; Hughes, 1994; El Albani et al., 2004; Kujau et al., 2013).

Most angiosperm pollen grains recorded from the earlier part of the Early Cretaceous (Valanginian/Hauterivian) are small, monosulcate or sometimes trichotomosulcate, finely reticulate to perforate, and show a distinct columellate structure. The genus *Clavatipollenites* and the species *C. hughesii* are among the most frequently mentioned taxa from this interval (e.g., Doyle, 1969; Gübeli et al., 1984; Burger, 1990, 1996; Trincão, 1990). One of the commonly cited records apparently includes several well-preserved angiosperm pollen grains reported from the Valanginian/Hauterivian of Italy. However, this record is documented only in an abstract without illustrations (Trevisan, 1988). Brenner (1996) published several finds from the Helez Formation of Israel of a probable Valanginian to Hauterivian age. However, the preservation of the figured specimens (Brenner, 1996, Figures 5.4 A–D) is too poor to support an angiosperm affinity; hence, Brenner's statement that the first angiosperms were represented by inaperturate grains cannot be corroborated. Some of the early records of angiosperm pollen are limited to the genus *Clavatipollenites*. A stratigraphically well constrained record exists for *C. hughesii* for the Valanginian of Morocco, where this form co-occurs with age-diagnostic dinoflagellate cysts, e.g., *Biorbifera johnewingii* and *Druggidium* spp. (Gübeli et al., 1984).

Based on the distribution of angiosperm pollen Hughes (1994) defined five phases for the palynological record of the Wealden of Southern England. The oldest phase (phase 0, Hauterivian) contains few angiosperm pollen grains; one of them might be assigned to the genus *Clavatipollenites* [Palaeotaxon Hauterivian-microtect of Hughes (1994)], another represents a tectate form with verrucate surface [Palaeotaxon Hauterivian-cactisulc of Hughes (1994)] and a third one is coarsely reticulate [Palaeotaxon—lacebee of Hughes (1994)]. Phase 1, probably of late Hauterivian age, includes a considerable number of monosulcate, reticulate morphotypes. Most of these grains [Retisulc group of Hughes (1994)] seem to fall in the morphological range of the *Retimonocolpites* complex or could be assigned to *Clavatipollenites* or *Tucanopollis* morphotypes [Palaeotaxon Barremian-ring of Hughes (1994)]. The increase in diversity of the angiosperm pollen between phase 0 and phase 1—taking place during the Hauterivian—is remarkable; it implies a considerable diversification within approximately 3.5 Ma or else it represents a wave of immigration from other areas. Additionally, Barremian pollen records suggest an already wide distribution of the angiosperms, including Gondwana with Africa (e.g., Doyle et al., 1977; Schrank and Mahmoud, 2002), South America (e.g., Regali and Viana, 1989) and Australia (e.g., Burger, 1990, 1996); Laurasia with North America (Burden, 1984), Europe (e.g., Hughes, 1994; Heimhofer et al., 2007), Eastern Russia (Markevitch, 1994) and China (e.g., Wang, 2000; Zhou et al., 2009); and show a considerable diversity and abundance in some areas (e.g., Egypt, Schrank and Mahmoud, 2002), (for additional references see also Eklund et al., 2004)

Based on close morphological similarities *Clavatipollenites* has been associated with the family of Chloranthaceae—namely with *Ascarina* (Couper, 1960; Doyle, 1969; Walker and Walker, 1984; Endress, 1987; Eklund et al., 2004). However, based on the detailed study of Hughes (1994) it seems that pollen grains determined as *Clavatipollenites* have to be regarded as a heterogeneous entity, probably including several groups of various affinity, e.g., Chloranthaceae, and probably related extinct groups (Pedersen et al., 1991) as well as *Austrobaileyales* (Endress and Honegger, 1980) thus including some representatives of the most basal families of the ANITA grade (including *Amborella*, Nymphaeales, Illiciales, *Trimenia*, and Austrobaileyales). However, *Clavatipollenites* represents the most commonly recorded pollen from the earlier part of the Early Cretaceous (Valanginian—early Hauterivian). Doyle (2012) suggested that these pollen grains represent the first angiosperm crown group fossils. So far the basal angiosperms of the ANITA grade are poorly represented in the early angiosperm pollen records. Most of these plants produce columellate and tectate pollen (Doyle, 2012). One possible early record from the early Hauterivian is represented by the tectate angiosperm pollen reported as Palaeotaxon Hauterivian-cactisulc (Hughes, 1994), which shows some resemblance with the pollen of *Amborella*. However, the majority of the pollen grains from the earlier part of the Lower Cretaceous (pre-Aptian) are semitectate and commonly show a well-developed reticulum. According to Doyle (2012) semitectate conditions with a distinctly reticulate sexine arose at the node connecting Austrobaileyales and mesangiosperms.

Evidence for pre-Cretaceous presence of angiosperms is difficult to assess. Some finds from the Jurassic announced with much public attention proved to be based on wrong ages (Sun et al., 1998; He et al., 2004, 2006) and the interpretations of other remains from the Jurassic (e.g., *Schmeissneria*) are controversial (Wang et al., 2007; van Konijnenburg-van Cittert et al., 2010; Wang, 2010; Friis et al., 2011; Doyle, 2012). Angiosperm-like pollen finds from this period are rare or questionable; the pollen reported from the Oxfordian of France (Cornet and Habib, 1992) are regarded here as contamination (see also Friis et al., 2011). *Clavatipollenites* has been repeatedly reported from the Jurassic (e.g., Pocock, 1962, 1970; Schulz, 1967; Vigran and Thusu, 1975; Abbink, 1998). For at least some of these grains Doyle et al. (1975) and Batten and Dutta (1997) proved a clear gymnospermous affinity. However, in routine palynological studies it might be difficult to distinguish small monosulcate grains with distinct angiospermous columellate structure from those with a clearly gymnospermous spongy alveolar structure.

Triassic records of angiosperms or angiosperm-like plants are discussed since the discovery of the famous leaf remains described as *Sanmiguelia* (Brown, 1956). Subsequent discoveries of reproductive structures of this plant and of angiosperm-like pollen in Late Triassic sediments nourished the debate about a Triassic origin of the group (Cornet, 1977, 1986, 1989a,b). Despite considerable attention the interpretation and attribution of the leaves and the reproductive structures *(Axelrodia)* of *Sanmiguelia* remain ambiguous (Friis et al., 2011; Doyle, 2012). Whereas some authors considered it as an angiosperm (Brown, 1956; Cornet, 1986, 1989a) others suggested an attribution to Ginkgophytes and rejected a possible relation to angiosperms (Crane, 1987; Doyle and Donoghue, 1993). Pollen grains associated with male organs show clear gymnospermous features. Recently, Doyle (2012) proposed that *Sanmiguelia* might represent a plant attached to the stem lineage of the angiosperms or a gymnosperm without closer relationship to angiosperms.

The affinity of the pollen group described as Crinopolles (including the genera *Tricrinopollis, Monocrinopollis, Dicrinopollis*, and *Zonacrinopollis*) from the Late Triassic (Carnian) of Virginia (Cornet, 1989b) continues to be a subject of controversy (e.g., Zavada, 2007; Friis et al., 2011; Doyle, 2012). These pollen grains show some clear angiospermous features (Cornet, 1989b); they are monosulcate and have a semitectate (reticulate) outer wall, which is connected by columellae to the inner wall (nexine). Other features, like the uniformly thick endexine, are more gymnosperm-like (Doyle and Hotton, 1991). Recently, it has been suggested that the Crinopolles group may represent plants on the angiosperm stem lineage (Doyle and Hotton, 1991; Doyle and Donoghue, 1993; Doyle, 2012). Since its original description this pollen group has been reported only sporadically (e.g., Litwin and Ash, 1993); it seems that its parent plants had an either temporally or regionally restricted distribution. *Cornetipollis reticulata* is another interesting pollen type from the Late Triassic that shows all the essential characteristics of angiosperm pollen (Pocock and Vasanthy, 1988). It is characterized by numerous exinal bands that are semitectate (reticulate and perforate) and are connected to the very thin inner layer (nexine) by columellae.

Middle Triassic angiosperm-like pollen grains have been published from drill cores from the Norwegian Barents Sea (Hochuli and Feist-Burkhardt, 2004). Previously these forms were repeatedly reported as *Retisulcites* sp. 1 and sp. 2 from Anisian and Ladinian sediments of exploration wells and well-calibrated cores (Hochuli et al., 1989; Vigran et al., 1998). Hochuli and Feist-Burkhardt (2004) attributed these pollen grains to angiosperms or to a new, unknown group of gymnosperms. These findings have received rather mixed attention. Despite distinct morphological differences (e.g., very thin nexine) Doyle (2012) associated them with the Crinopolles group. Other authors accepted them as clear evidence for angiosperms (Zavada, 2007; Clarke et al., 2011), or as interesting evidence to consider (Taylor et al., 2009), whereas others questioned the attribution to angiosperms (Maheshwari, 2007) or simply ignored them (Friis et al., 2011).

The pollen grains here described add to the considerable diversity already documented from the Middle Triassic of the Barents Sea (Hochuli and Feist-Burkhardt, 2004). In the Barents Sea they were found in palynological assemblages with high representation of spores reflecting rather humid environments (Hochuli and Vigran, 2010). During the Anisian the sections from Northern Switzerland were located about 20°N. The angiosperm-like pollen grains come from an interval consisting of evaporites with intercalated thin layers of sand and siltstones. Here, the lithology as well as the associated palynofloras, characterized by pronounced dominance of the *Triadispora* group, known to represent xerophytic elements (Kürschner and Herngreen, 2010), as well as the scarcity of spores, suggest a dry climate for this interval.

Representatives of the *Afropollis* group are common elements of Cretaceous low latitudinal spore-pollen assemblages. Originally described by Doyle et al. (1982) as early angiosperm pollen and showing some angiospermous features, they are still considered as such by many authors (e.g., Krassilov and Schrank, 2011; Coiffard et al., 2012). Their attribution to angiosperms was called into question by the discovery of an (unpaired) pollen sac containing *in situ Afropollis* (Friis et al., 2011). Consequently, we consider them here as representatives of a so far unknown group of gymnosperms with singular pollen morphology. The known stratigraphic range of the *Afropollis* group covers the interval between the Barremian (e.g., Penny, 1989; Schrank and Mahmoud, 2002) and the middle Cenomanian (e.g., Schrank and Ibrahim, 1995). Its main distribution lies between the late Barremian or early Aptian and the lower Cenomanian (Doyle et al., 1982; Doyle, 1992). Thus, similar to the angiosperms, our record of this group from the Middle Triassic opens another observation gap of over 100 Ma.

## Conclusions

Assessing the appearance of the angiosperms by fossil pollen grains is a rather difficult issue. However, according to Zavada (1984a) the combined characteristics of the described pollen (small, monosulcate, columellate, semitectate–reticulate) grains exhibit a full inventory of angiosperm pollen synapomorphies and probably also express the corresponding function, e.g., self-incompatibility (Zavada, 1984b). However, according to Doyle (2012) the early appearance of columellate reticulate pollen poses a problem for connecting them with the stem lineage or the most basal crown lineages. Based on molecular data the basal groups of angiosperms produce pollen grains with a continuous tectum and the semitectate, reticulate conditions developed at the node connecting Austrobaileyales and mesangiosperms.

Interpretation of the molecular evidence for the origin of flowering plants is controversial depending on the data and the calculation methods. Estimates for the origin of flowering plants range from the late Early Permian (275 Ma) to the Late Triassic (221.5 Ma) or Early Jurassic (193.8 Ma) (Magallón, 2010; Magallón et al., 2013). Other authors suggest a Late Triassic age (228–217 Ma) (Smith et al., 2010) or give a Jurassic age range (183–147 Ma) (Bell et al., 2010). Thus there are some calculations that would corroborate the first fossil evidence from the Middle Triassic.

If we accepted the monosulcate pollen from the Middle and Late Triassic as evidence for a pre-Cretaceous origin of crown group angiosperms the lack of fossil records throughout the Jurassic would remain difficult to explain. Some authors gave ecological reasons for this gap, such as that the early angiosperms evolved in upland environments, far from the potential sedimentary basins (Axelrod, 1952, 1970). From the present distribution and the physiology of extant basal angiosperms Feild et al. (2004, 2009) concluded that their ancestors must have lived in dark, wet and disturbed understory habitats. The possibly rare occurrences of such habitats in the Jurassic were used to explain the lack of fossil records. However, if the Triassic angiosperm-like pollen grains would represent an angiosperm crown group our evidence that they lived in a wide range of habitats, including arid environments, would contradict the hypothesis that early crown group angiosperms were restricted to wet understory habitats (Feild et al., 2004, 2009) and would make it still more difficult to explain the Jurassic gap in their record.

Compared to the early records in the Cretaceous (Valanginian—early Hauterivian) the diversity of the pollen grains described from the Triassic is rather high and their occurrence in different environments also suggests a high adaptability. Additionally, their morphology appears to be closer to the Cretaceous morphotypes of *Retimonocolpites*, thus to the phases 1–4 of the morphological succession of Hughes (1994), implying that there is no evident relationship between the pollen grains known from the Middle Triassic and the oldest Cretaceous angiosperm pollen (phase 0 of Hughes, 1994). Considering the hundred million year gap in the record as well as morphological differences to the earliest Cretaceous we suggest that these pollen grains most likely represent stem relatives of the angiosperms. Thus, the monosulcate, columellate conditions of the pollen might represent an ancestral feature, expressed at different stages in the evolution of the flowering plants, or alternatively, these features would represent analogues and were independently developed by an unrelated, and so far unknown, group of gymnosperms. However, since this group produced a wide variety of monosulcate and reticulate—thus angiosperm-like—pollen grains and could adapt to various environments, we expect that they may be found in other areas and in other stratigraphic and environmental contexts. As for the *Afropollis* group we have to await discoveries of the corresponding megafossils to learn more about the morphology and relationship of the parent plants of the pollen grains here described.

### Conflict of interest statement

The authors declare that the research was conducted in the absence of any commercial or financial relationships that could be construed as a potential conflict of interest.

## References

[B1] Abbink O. A. (1998). Palynological Investigations in the Jurassic of the North Sea Region. Ph.D. thesis. Utrecht: Universiteit Utrecht, 192

[B2] Axelrod D. I. (1952). A theory of angiosperm evolution. Evolution 6, 29–60 10.2307/2405502

[B3] Axelrod D. I. (1970). Mesozoic paleogeography and early angiosperm history. Bot. Rev. 36, 277–319 10.1007/BF02858880

[B4] Batten D. J. Dutta R. J. (1997). Ultrastructure of exine of gymnospermous pollen grains from the Jurassic and basal Cretaceous in Northwest Europe and implications for botanical relationships. Rev. Palaeobot. Palynol. 99, 25–45 10.1016/S0034-666700036-5

[B5] Bell C. D. Soltis D. E. Soltis P. S. (2010). The age and diversification of the angiosperms re-revisited. Am. J. Bot. 97, 1296–1303 10.3732/ajb.0900346 21616882

[B6] Brenner G. J. (1996). “Evidence for the earliest stage of angiosperm pollen evolution: a paleoequatorial section from Israel,” in Flowering Plant Origin, Evolution and Phylogeny, eds Taylor D. W. Hickey L. J. (New York, NY: Chapman and Hall), 91–115 10.1007/978-0-585-23095-5_5

[B7] Brown R. W. (1956). Palmlike plants from the Dolores Formation (Triassic) in southwestern Colorado. US Geol. Surv. Prof. Pap. 274H, 205–209

[B8] Burger D. (1990). Early angiosperms from Queensland, Australia. Rev. Palaeobot. Palynol. 65, 153–161 10.1016/0034-6667(90)90066-R

[B9] Burger D. (1996). Mesozoic palynomorphs from the North West Shelf, offshore Western Australia. Palynology 20, 49–103 10.1080/01916122.1996.9989470

[B10] Burden E. T. (1984). “Terrestrial palynomorph biostratigraphy of the lower part of the Mannville Group (Lower Cretaceous) Alberta and Montana,” in The Mesozoic of Middle North America, Vol. 9, eds Stott D. F. Glass D. J. (Calgary, AB: Canadian Society of Petroleum Geologists), 249–269

[B11] Coiffard C. Gomez B. Daviero-Gomez V. Dilcher D. L. (2012). Rise to dominance of angiosperm pioneers in European Cretaceous environments. Proc. Natl. Acad. Sci. U.S.A. 109, 20955–20959 10.1073/pnas.1218633110 23213256 PMC3529080

[B12] Clarke J. T. Warnock R. C. M. Donoghue P. C. J. (2011). Establishing a time-scale for plant evolution. New Phytol. 1–36 10.1111/j.1469-8137.2011.03794.x 21729086

[B13] Cornet B. (1977). “Angiosperm-like pollen with tectate-columellate wall structure from the Upper Triassic (and Jurassic) of the Newark Supergroup, USA,” in American Association of Stratigraphic Palynologists 10th Annual Meeting, (Tulsa), Abstracts, 8–9

[B14] Cornet B. (1986). The leaf venation and reproductive structures of a Late Triassic angiosperm, *Sanmiguelia lewisii*. Evol. Theory 7, 231–309

[B15] Cornet B. (1989a). The reproductive morphology and biology of *Sanmiguelia lewisii*, and its bearing on angiosperm evolution in the Late Triassic. Evol. Trends Plants 3, 25–51

[B16] Cornet B. (1989b). Late Triassic angiosperm-like pollen from the Richmond Rift Basin of Virginia, U.S.A. Palaeontographica Abt. B 213, 37–87

[B17] Cornet B. (1996). “A new gnetophyte from the Late Carnian (Late Triassic) of Texas and its bearing on the origin of the angiosperm carpel and stamen,” in Flowering Plant Origin, Evolution and Phylogeny, ed Taylor D. W. Hickey L. J. (New York, NY: Chapman and Hall), 32–67 10.1007/978-0-585-23095-5_3

[B18] Cornet B. Habib D. (1992). Angiosperm-like pollen from the ammonite-dated Oxfordian (Upper Jurassic) of France. Rev. Palaeobot. Palynol. 71, 269–294 10.1016/0034-666790167-F

[B19] Couper R. A. (1960). New Zealand Mesozoic and Cainozoic plant microfossils. N.Z. Geol. Surv. Palaeontol. Bull. 32, 1–87

[B20] Crane P. R. (1987). Review of Cornet 1986. Evol. Theory 7, 231–309

[B21] Crane P. R. Lidgard S. (1989). Angiosperm diversification and palaeolatitudinal gradients in Cretaceous floristic diversity. Science 246, 675–678 10.1126/science.246.4930.675 17833420

[B22] Dörhöfer G. (1977). Palynologie und Stratigraphie der Bückeberger-Formation (Berriasium-Valanginium) in der Hilsmulde (NW-Deutschland). Geol. Jb. A 42, 3–122

[B23] Dörhöfer G. Norris G. (1977). Discrimination and correlation of highest Jurassic and lowest Cretaceous terrestrial palynofloras in north-west Europe. Palynology 1, 79–93 10.1080/01916122.1977.9989151

[B24] Doyle J. A. (1969). Cretaceous angiosperm pollen of the Atlantic Coastal Plain and its evolutionary significance. J. Arnold Arbor. 50, 1–35

[B25] Doyle J. A. (1983). Evidence for Berriasian age of basal Potomac Group sediments, Crisfield well, eastern Maryland. Pollen Spores 25, 499–530

[B26] Doyle J. A. (1992). Revised palynological correlations of the lower Potomac Group (USA) and the Cocobeach sequence of Gabon (Barremian-Aptian). Cretac. Res. 13, 337–349 10.1016/0195-667190039-S

[B27] Doyle J. A. (2012). Molecular and fossil evidence on the origin of angiosperms. Annu. Rev. Earth Planet. Sci. 40, 301–326 10.1146/annurev-earth-042711-105313

[B28] Doyle J. A. Donoghue M. J. (1993). Phylogenies and angiosperm diversification. Paleobiology 19, 141–167

[B29] Doyle J. A. Hotton C. L. (1991). “Diversification of early angiosperm pollen in a cladistic context,” in Pollen and Spores: Patterns of Diversification, eds Blackmore S. Barnes S. H. (Oxford: Clarendon), 169–195

[B30] Doyle J. A. Van Campo M. Lugardon B. (1975). Observations on exine structure of *Eucommiidites* and Lower Cretaceous angiosperm pollen. Pollen Spores 17, 429–486

[B31] Doyle J. A. Biens P. Doerenkamp A. Jardiné S. (1977). Angiosperm pollen from the pre-Albian Lower Cretaceous of equatorial Africa. Bull. Cent. Rech. Explor. Prod. Elf-Aquitaine 1, 451–473

[B32] Doyle J. A. Jardiné S. Doerenkamp A. (1982). *Afropollis*, a new genus of early angiosperm pollen, with notes on the Cretaceous palynostratigraphy and paleoenvironments of Northern Gondwana. Bull. Cent. Rech. Explor. Prod. Elf-Aquitaine 6, 39–117

[B33] El Albani A. Fürsich F. T. Colin J.-P. Meunier A. Hochuli P. A. Martín-Closas C. (2004). Palaeoenvironmental reconstruction of the basal Cretaceous vertebrate bearing beds in the Northern part of the Aquitaine Basin (SW France): sedimentological and geochemical evidence. Fazies 50, 195–215 10.1007/s10347-004-0017-6

[B34] Eklund H. Doyle J. A. Herendeen P. S. (2004). Morphological phylogenetic analysis of living and fossil Chloranthaceae. Int. J. Plant Sci. 165, 107–151 10.1086/380987

[B35] Endress P. K. (1987). The Chloranthaceae: reproductive structures and phylogenetic position. Bot. Jahrb. Syst. Pflanzengeschichte Pflanzengeographie 109, 153–226

[B36] Endress P. K. Honegger R. (1980). The pollen of Austrobaileyaceae and its phylogenetic significance. Grana 19, 177–182 10.1080/00173138009425001

[B37] Feild T. S. Arens N. C. Doyle J. A. Dawson T. E. Donoghue M. J. (2004). Dark and disturbed: a new image of early angiosperm ecology. Paleobiology 30, 82–107 10.1666/0094-8373030<0082:DADANI>2.0.CO;2

[B38] Feild T. S. Chatelet D. S. Brodribb T. J. (2009). Ancestral xerophobia: a hypothesis on the whole plant ecophysiology of early angiosperms. Geobiology 7, 237–264 10.1111/j.1472-4669.2009.00189.x 19260972

[B39] Feist-Burkhardt S. Pross J. (1999). Morphological analysis and description of Middle Jurassic dinoflagellate cyst marker species using confocal laser scanning microscopy, digital optical microscopy, and conventional light microscopy. Bull. Cent. Rech. Explor. Prod. Elf-Aquitaine, 22, 103–145

[B40] Friis E. M. Crane P. R. Pedersen K. R. (2011). Early Flowers and Angiosperm Evolution. Cambridge: Cambridge University Press 10.1017/CBO9780511980206

[B41] Frohlich M. W. Chase M. W. (2007). After a dozen years of progress the origin of angiosperms is still a great mystery. Nature 450, 1184–1189 10.1038/nature06393 18097399

[B42] Gübeli A. Hochuli P. A. Wildi W. (1984). Lower Cretaceous turbiditic sediments from the Central Rif chain (northern Morocco). Palynology, stratigraphy and palaeogeographic setting. Geol. Rundschau 73, 1081–1114 10.1007/BF01820889

[B43] He H. Y. Wang X. L. Zhou Z. H. Wang F. Boven A. Shi G. H. (2004). Timing of the Jiufotang Formation (Jehol Group) in Liaoning, northeastern China and its implications. Geophys. Res. Lett. 31, L12605, 10.1029/2004GL019790

[B44] He H. Y. Wang X. L. Zhou Z. H. Jin F. Wang F. Yang L. K. (2006). 40Ar/39Ar dating of Lujiatun Bed (Jehol Group) in Liaoning, northeastern China. Geophys. Res. Lett. 33 L04303, 10.1029/2005GL025274

[B45] Heimhofer U. Hochuli P. A. Burla S. Dinis J. Weissert H. (2005). Timing of Early Cretaceous angiosperm diversification and possible links to major palaeoenvironmental change. Geology 33, 141–144 10.1130/G21053.1

[B46] Heimhofer U. Hochuli P. A. Burla S. Weissert H. (2007). New records of Early Cretaceous angiosperm pollen from Portuguese coastal deposits: implications for the timing of the early angiosperm radiation. Rev. Palaeobot. Palynol. 144, 39–76 10.1016/j.revpalbo.2005.09.006

[B47] Hickey L. J. Doyle J. A. (1977). Early Cretaceous fossil evidence for angiosperm evolution. Bot. Rev. 43, 1–104 10.1007/BF02860849

[B48] Hochuli P. A. Colin J. P. Vigran J. O. (1989). “Triassic biostratigraphy of the Barents Sea Area,” in Correlation in Hydrocarbon Exploration, ed Collinson J. (Graham and Trotman), 131–153 10.1007/978-94-009-1149-9_12

[B49] Hochuli P. A. Feist-Burkhardt S. (2004). A boreal early cradle of Angiosperms. Angiosperm-like pollen from the Middle Triassic of the Barents Sea (Norway). J. Micropalaeontol. 23, 97–104 10.1144/jm.23.2.97

[B50] Hochuli P. A. Vigran J. O. (2010). Climate variations in the Boreal Triassic – inferred from palynological records from the Barents Sea. Palaeogeogr. Palaeoclimatol. Palaeoecol. 290, 20–42 10.1016/j.palaeo.2009.08.013

[B51] Hochuli P. A. Rebetez D. Schneebeli-Hermann E. Ramseyer K. (2012). “Middle Triassic of the Weiach borehole – results from palynology and isotope chemostratigraphy,” in 10th Swiss Geoscience Meeting, Abstract, Vol. 7, (Bern), 168

[B52] Hughes N. F. (1994). The Enigma of Angiosperm Origins. Cambridge, UK: Cambridge University Press

[B53] Krassilov V. Schrank E. (2011). New Albian macro- and palynoflora from the Negev (Israel) with description of a new gymnosperm morphotaxon. Cretac. Res. 32, 13–29 10.1016/j.cretres.2010.10.001

[B54] Kürschner W. M. Herngreen W. (2010). “Triassic palynology of central and northwestern Europe: a review of palynofloral diversity patterns and biostratigraphic subdivisions,” in The Triassic Timescale, Vol. 334, ed Lucas S. G. (London: Geological Society London Special Publications), 263–283 10.1144/SP334.11

[B55] Kujau A. Heimhofer U. Hochuli P. A. Pauly S. Morales C. Adatte T. (2013). Reconstructing Valanginian (Early Cretaceous) mid-latitude vegetation and climate dynamics based on spore-pollen assemblages. Rev. Palaeobot. Palynol. 197, 50–69 10.1016/j.revpalbo.2013.05.003

[B56] Litwin R. J. Ash S. R. (1993). Revision of the biostratigraphy of the Chatham Group (Upper Triassic), Deep River basin, North Carolina, USA. Rev. Palaeobot. Palynol. 77, 75–95 10.1016/0034-666790057-2

[B57] Magallón S. (2010). Using fossils to break long branches in molecular dating: a comparison of relaxed clocks applied to the origin of angiosperms. Syst. Biol. 59, 384–399 10.1093/sysbio/syq027 20538759

[B58] Magallón S. Hilu K. W. Quandt D. (2013). Land plant evolutionary timelines: Gene effects are secondary to fossil constraints in relaxed clock estimation of age and substitution rates. Am. J. Bot. 100, 556–573 10.3732/ajb.1200416 23445823

[B59] Maheshwari H. K. (2007). Deciphering angiosperm origins. Curr. Sci. 92, 606–611

[B60] Markevitch V. S. (1994). Palynological zonation of the continental Cretaceous and lower Tertiary of eastern Russia. Cretac. Res. 15, 165–177 10.1006/cres.1994.1008

[B61] Matter A. Peters T. Bläsi H.-R. Meyer J. Ischi H. Meyer Ch. (1988). Sondierbohrung Weiach – Geologie (Textband). Nagra Technischer Bericht NTB 86-01, (Baden), 470

[B62] Muller J. (1981). Fossil pollen records of extant angiosperms. Bot. Rev. 47, 1–142 10.1007/BF02860537

[B63] Norris G. (1969). Miospores from the Purbeck Beds and marine Upper Jurassic of southern England. Palaeontology 12, 574–620

[B64] Pedersen K. R. Crane P. R. Drinnan A. N. Friis E. M. (1991). Fruits from the mid-Cretaceous of North America with pollen grains of the *Clavatipollenites* type. Grana 30, 577–590 10.1080/00173139109427816

[B65] Penny J. H. J. (1989). New Early Cretaceous forms of the angiosperm pollen genus *Afropollis* from England and Egypt. Rev. Palaeobot. Palynol. 58, 289–299 10.1016/0034-666790089-4

[B66] Peters T. Matter A. Bläsi H.-R. Isenschmid Ch. Klebloth P. Meyer Ch. Meyer J. (1989). Sondierbohrung Leuggern – Geologie (Textband). Nagra Technischer Bericht NTB 86-05, (Baden), 250

[B67] Pocock S. A. J. (1962). Microfloral analysis and age determination of strata at the Jurassic-Cretaceous boundary in the western Canada plains. Palaeontographica Abt. B 111, 1–95

[B68] Pocock S. A. J. (1970). Palynology of the Jurassic sediments of western Canada. Part 1. Terrestrial species. Palaeontographica Abt. B 130, 12–72

[B69] Pocock S. A. J. Vasanthy G. (1988). *Cornetipollis reticulata*, a new pollen with angiospermid features from the upper Triassic (Carnian) sediments of Arizona (U.S.A.), with notes on *Equisetosporites*. Rev. Palaeobot. Palynol. 55, 337–356 10.1016/0034-666790092-9

[B70] Regali M. S. P. Viana C. F. (1989). Late Jurassic–Early Cretaceous in Brazilian Sedimentary Basins: Correlation with an International Standard Stratigraphic Scale. Rio de Janeiro: Petrobrás Petroleo Brasileiro, S. A

[B71] Schindelin J. Arganda-Carreras I. Frise E. Kaynig V. Longair M. Pietzsch T. (2012). Fiji: an open-source platform for biological-image analysis, Nat. Methods 9, 676–682 10.1038/nmeth.2019 22743772 PMC3855844

[B72] Schrank E. Ibrahim M. I. A. (1995). Cretaceous (Aptian – Maastrichtian) palynology of foraminifera-dated wells (KRM-1, AG-18) in Northwestern Egypt. Berl. Geowissenschaftl. Abh. Reihe A Geol. Paläontol. 177, 1–44

[B73] Schrank E. Mahmoud M. S. (2002). Barremian angiosperm pollen and associated palynomorphs from the Dakhla Oasis Area, Egypt. Palaeontology 45, 33–56 10.1111/1475-4983.0022611042331

[B74] Schulz E. (1967). Sporenpaläontologische Untersuchungen in rhätoliassischer Schichten im Zentralteil des Germanischen Beckens. Paläontol. Abh. Abt. B 2, 541–633

[B75] Smith S. A. Beaulieu J. M. Donoghue M. J. (2010). An uncorrelated relaxed-clock analysis suggests an earlier origin for flowering plants. Proc. Natl. Acad. Sci. U.S.A. 107, 5897–5902 10.1073/pnas.1001225107 20304790 PMC2851901

[B76] Soltis D. E. Moore M. J. Burleigh J. G. Bell C. D. Soltis P. S. (2010). Assembling the angiosperm tree of life: progress and future prospects. Ann. Mo. Bot. Gard. 97, 514–526 10.3417/2009136

[B77] Soltis D. E. Smith S. A. Cellinese N. Wurdack K. J. Tank D. C. Brockington S. F. (2011). Angiosperm phylogeny: 17 genes, 640 taxa. Am. J. Bot. 98, 704–730 10.3732/ajb.1000404 21613169

[B78] Sun G. Dilcher D. L. Zheng S. Zhou Z. (1998). In search of the first flower: a Jurassic angiosperm, *Archaefructus*, from northeast China. Science 282, 1692–1695 10.1126/science.282.5394.1692 9831557

[B79] Staplin F. L. (1982). “Determination of thermal alteration index from color of exinite (pollen, spores),” in How to Assess Maturation and Palaeotemperatures, Vol. 7, ed. Staplin F. L. (Society of Economic Paleontology and Mineralogy) 7–11

[B80] Stuessy T. F. (2004). A transitional-combinational theory for the origin of angiosperms. Taxon 53, 3–16 10.2307/413548423019892

[B81] Taylor T. N. Taylor E. L. Krings M. (2009). Paleobotany. The Biology and Evolution of Fossil Plants. Burlington; London; San Diego; New York: Elsevier/Academic Press Inc

[B82] Traverse A. (2007). Paleopalynology. Dordrecht: Springer

[B83] Trevisan L. (1988). “Angiospermous pollen (monosulcatetrichotomosulcate phase) from very early Lower Cretaceous of Southern Tuscany (Italy): some aspects,” in Proc. 7th Int. Palynol. Congr. Brisb. Aust. Abstr. 165. (Amsterdam: Elsevier).

[B84] Trincão P. R. P. (1990). “Esporos e pólenes do Cretácio Inferior (Berriasiano-Aptiano) de Portugal: Paleontologia e Biostratigrafia. PhD Thesis. Lisbon: Universidade Nova de Lisboa, 1–312

[B85] van Konijnenburg-van Cittert J. H. A. (2010). The Early Jurassic male ginkgoalean inflorescence *Stachyopitys preslii* Schenk and its in situ pollen. Scripta Geol. 7, 141–149

[B86] Vigran J. O. Mangerud G. Mork A. Bugge T. Weitschat W. (1998). Biostratigraphy and sequence stratigraphy of the Lower and Middle Triassic deposits from the Svalis Dome, central Barents Sea, Norway. Palynology 22, 89–141 10.1080/01916122.1998.9989505

[B87] Vigran J. O. Thusu B. (1975). Illustrations and Distribution of the Jurassic Palynomorphs of Norway: Illustrations of Norwegian Microfossils. Trondheim: NTNFkontinentalsokkelkontor

[B88] Walker J. W. Walker A. G. (1984). Ultrastructure of Lower Cretaceous angiosperm pollen and the origin and early evolution of flowering plants. Ann. Mo. Bot. Gard. 71, 464–521 10.2307/2399035

[B89] Wang X. (2000) “Pollen record of early angiosperms in NE China,” in Palynofloras and Palynomorphs of China ed Song Z. (Hefei: Press University Science and Technology China), 96–100

[B90] Wang X. (2009). “New fossils and new hope for the origin of angiosperms,” in Evolutionary Biology: Concepts, Modelling, and Application, ed Pontarotti P. (Berlin; Heidelberg: Springer-Verlag), 51–70 10.1007/978-3-642-00952-5_3

[B91] Wang X. (2010). *Schmeissneria*: an angiosperm from the Early Jurassic. J. Syst. Evol. 48, 326–335 10.1111/j.1759-6831.2010.00090.x17284326

[B92] Wang X. Duan S. Y. Geng B. Y. Cui J. Z. Yang Y. (2007). *Schmeissneria*: a missing link to angiosperms. BMC Evol. Biol. 7:14 10.1186/1471_2148-7-14 17284326 PMC1805421

[B93] Zavada M. S. (1984a). Angiosperm origins and evolution based on dispersed fossil pollen ultrastructure. Ann. Mo. Bot. Gard. 71, 440–459 10.2307/2399034

[B94] Zavada M. S. (1984b). The relation between pollen exine sculpturing and self-incompatibility mechanisms. Plant Syst. Evol. 147, 63–78 10.1007/BF00984580

[B95] Zavada M. S. (2007). The identification of fossil angiosperm pollen and its bearing on the time and place of the origin of angiosperms. Plant Syst. Evol. 263, 1–2, 117–134. 10.1007/s00606-006-0495-9

[B96] Zheng S. L. Wang X. (2010). An undercover angiosperm from the Jurassic of China. Acta Geol. 84, 895–902 10.1111/j.1755-6724.2010.00252.x

[B97] Zhou S. Zhou L. Wang W. Wu Y. Yang X. (2009). Cretaceous Palynostratigraphy, with Emphasis on Angiosperm Pollen Grains and their Evolution in Jiangsu Province, China. Nanjing: Sinopec, Huadon Company (in Chinese with English abstract).

